# Intracerebral gadolinium deposition following blood–brain barrier disturbance in two different mouse models

**DOI:** 10.1038/s41598-023-36991-8

**Published:** 2023-06-22

**Authors:** M. L. Kromrey, S. Oswald, D. Becher, J. Bartel, J. Schulze, H. Paland, T. Ittermann, S. Hadlich, J. P. Kühn, S. Mouchantat

**Affiliations:** 1 Department of Diagnostic Radiology and Neuroradiology, University Medicine Greifswald, Ferdinand-Sauerbruch-Straße, 17475 Greifswald, Germany; 2 Institute of Pharmacology and Toxicology, Rostock University Medical Center, Rostock, Germany; 3 Department of Microbial Proteomics, Institute of Microbiology, University of Greifswald, Greifswald, Germany; 4 Department of Neurology, University Medicine Greifswald, Greifswald, Germany; 5 Department of Pharmacology/C_DAT, University Medicine Greifswald, Greifswald, Germany; 6 Department of Neurosurgery, University Medicine Greifswald, Greifswald, Germany; 7 Institute for Community Medicine, University Medicine Greifswald, Greifswald, Germany; 8 Institute and Policlinic of Diagnostic and Interventional Radiology, University Hospital Carl Gustav Carus, Technische Universität Dresden, Dresden, Germany

**Keywords:** Imaging, Magnetic resonance imaging, Medical research, Neurology

## Abstract

To evaluate the influence of the blood–brain barrier on neuronal gadolinium deposition in a mouse model after multiple intravenous applications of the linear contrast agent gadodiamide. The prospective study held 54 mice divided into three groups: healthy mice (A), mice with iatrogenic induced disturbance of the blood–brain barrier by glioblastoma (B) or cerebral infarction (C). In each group 9 animals received 10 iv-injections of gadodiamide (1.2 mmol/kg) every 48 h followed by plain T1-weighted brain MRI. A final MRI was performed 5 days after the last contrast injection. Remaining mice underwent MRI in the same time intervals without contrast application (control group). Signal intensities of thalamus, pallidum, pons, dentate nucleus, and globus pallidus-to-thalamus and dentate nucleus-to-pons ratios, were determined. Gadodiamide complex and total gadolinium amount were quantified after the last MR examination via LC–MS/MS and ICP-MS. Dentate nucleus-to-pons and globus pallidus-to-thalamus SI ratios showed no significant increase over time within all mice groups receiving gadodiamide, as well as compared to the control groups at last MR examination. Comparing healthy mice with group B and C after repetitive contrast administration, a significant SI increase could only be detected for glioblastoma mice in globus pallidus-to-thalamus ratio (p = 0.033), infarction mice showed no significant SI alteration. Tissue analysis revealed significantly higher gadolinium levels in glioblastoma group compared to healthy (p = 0.013) and infarction mice (p = 0.029). Multiple application of the linear contrast agent gadodiamide leads to cerebral gadolinium deposition without imaging correlate in MRI.

## Introduction

Since their first description in 1984^1^, Gadolinium-based contrast agents (GBCAs) are used million-fold in clinical practice and research projects worldwide. Due to its toxicity, gadolinium cannot be administered in its free form, but in chelate binding—linear or macrocyclic—which defines the general properties and functions of the complex^2,3^. Although GBCA application was associated with a rare but fatal disease—nephrogenic systemic fibrosis (NSF)—in patients with renal dysfunction, the usage in subjects with sufficient renal excretion was considered safe until recently^4,5^, as they are rapidly eliminated from the body^6^. However, high signal intensities found on unenhanced MR images in the globus pallidus and dentate nucleus suggested that gadolinium may cross the blood–brain barrier (BBB) and accumulate in neuronal tissue. The cerebral deposition of gadolinium as a cause of such T1 abnormalities was first reported by Kanda et al. and McDonald et al.^7–10^. Remarkably, these findings occurred independently from renal or liver function, and were correlated with the number of previous GBCA exposures. Furthermore, osseous accumulation was detected after repeated GBCA administration even in subjects with normal renal function^11,12^ and it was proposed that the bone may serve as a reservoir for free gadolinium^13^.

In the literature there exists, from time to time, some inconsistency regarding the appropriate terminology of residual gadolinium. According to reviews by Robert et al.^14^ and Le Fur et al.^15^ “retention” means retained gadolinium species with a slow elimination over time, whereas “deposition” should be used where no excretion occurs. Both terms do not, however, specify the different chemical forms of the substance (that is gadolinium salts like GdPO4, intact soluble GBCA, or soluble macromolecular complexes).

Accumulation is supposedly dependent on its chemical structure, as it was observed almost exclusively for linear chelates^16,17^. This process is most likely based on a rapid release of free gadolinium compared to macrocyclic agents, which appear to be more stable^3,7,17,18^. Consistently, Tweedle et al.^19^ showed different extents of gadolinium presence according to the type of chelate in rats and mice: gadodiamide (Omniscan®; linear, non-ionic) > gadopentetate dimeglumine (Magnevist®; linear, ionic) ≈ gadoterate meglumine (Dotarem®; macrocyclic, ionic) ≈ gadoteridol (ProHance®; macrocyclic, ionic). These findings were confirmed in humans by Kanda et al. as well as by Radbruch et al., revealing hyperintensities on MR T1-weighted images in the dentate nucleus after gadopentetate dimeglumine administration, but not following gadoteridol^16^ or gadoterate meglumine^17^. Although gadolinium presence within cerebral tissue was also chemically confirmed^8,10^, a histologic change could not be detected^20^.

The chemical analysis of possible effects of contrast agents on the brain is, for obvious reasons, difficult in humans. One possibility are tissues gained by autopsy or after surgical resection, for instance after tumor resection. However, an exact temporal correlation, led alone short-term investigation of neuronal changes proves to be impossible. Although imaging and autopsy analyses proofed, that gadolinium is able to penetrate the blood–brain barrier and deposits in certain brain structures, the mechanism of neuronal gadolinium accumulation still remains unclear. A neurotoxic effect, which could be associated with gadolinium exposition, was not found to date leaving the clinical relevance unclear. As an alternative approach, animal experiments enable specific quantitative and temporal analyses, always under the prerequisite of the 3 R rule to ensure an ethical use of animals in testing—replacement (use of alternative methods), reduction (use of fewest animals possible) and refinement (enhance animal welfare). By translating results to human level, animal experience may help to improve clinical practice in terms of optimized efficacy and safety of drugs.

In the presented study we relied on different experimental mouse models of blood–brain barrier disturbance, by which we investigated intra-cerebral gadolinium accumulation after intravenous injection of the linear contrast agent gadodiamide via MR imaging and quantitative laboratory analysis.

## Materials and methods

### Study design

**Figure 1:**
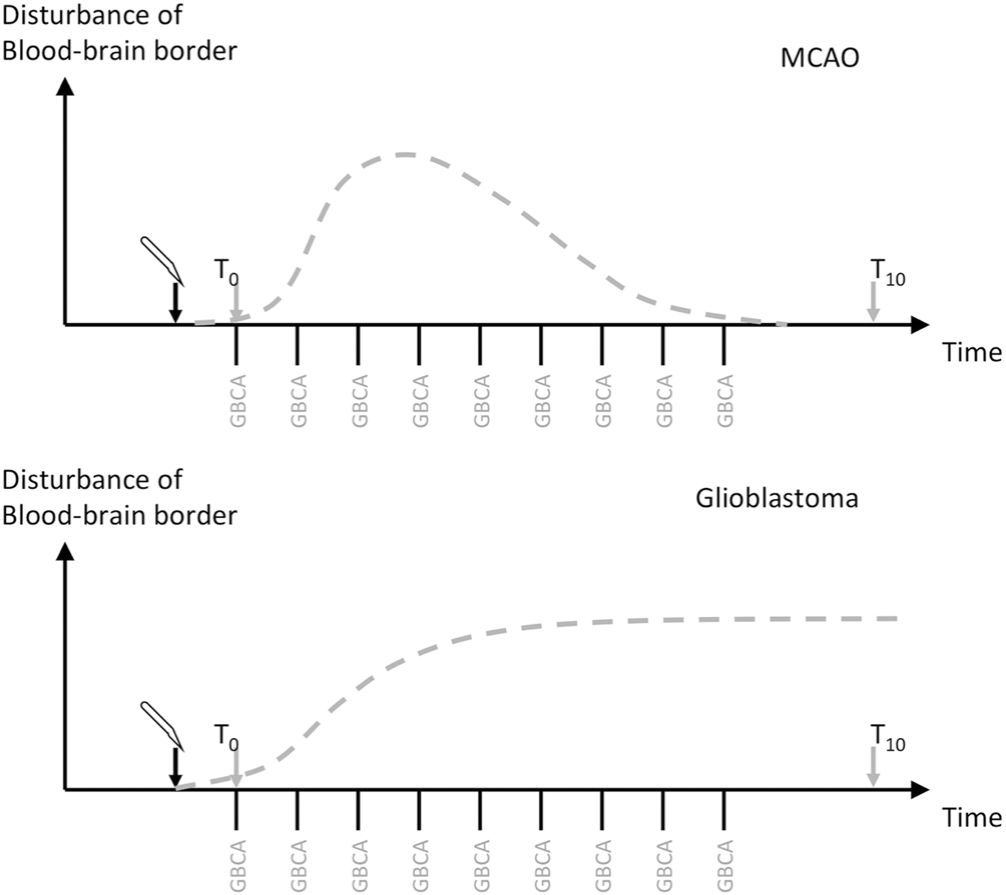
Experimental scheme for mouse groups B (glioblastoma) and C (infarction, MCAO) showing the course of blood–brain barrier disturbance over time.

The study protocol included a total of 54 mice divided into three groups: healthy mice (group A, n = 18) and mice with iatrogenic induced disturbance of the blood–brain barrier function by either glioblastoma (group B, n = 18) or middle cerebral arterial occlusion (MCAO, group C, n = 18). Both are approved animal disease models for imaging blood–brain barrier disturbance in mice^21^, with the difference that glioblastoma causes an increasing blood–brain barrier permeability, whereas MCAO leads only to a transient BBB disturbance^22^ (Fig. 1).

In each group 9 animals received 10 intravenous injections of the linear contrast agent gadodiamide (Omniscan™, GE Healthcare Buchler & Co, Germany) at a dose of 1.2 mmol/kg body weight every 48 h followed by plain T1-weighted brain magnetic resonance imaging (MRI). In mice, 1.2 mmol/kg dose is considered equivalent to the fourfold usual human dose of 0.1 mmol/kg after adjusting for body surface area^23^. In both groups with BBB disturbance, the first contrast agent injection was conducted 48 h after experimental stroke induction or glioblastoma cell implantation, respectively. A final MRI was performed 5 days after the last contrast injection. The remaining mice underwent MRI examinations in the same time intervals without prior contrast agent application and thereby served as control.

### Animals

Nine weeks old male C57BI6 mice (Charles-River Sulzfeld, Germany) were kept under standardized conditions (12 h light/dark cycle, room temperature 22 ± 1 °C, humidity 50–60%). Mice were euthanized under isoflurane anesthesia by cervical dislocation and the brain was removed from the skull to assess gadodiamide and gadolinium content. The samples were stored at − 80 °C for further use.

Animal experiments were approved by the supervisory Authority (LALLF—Landesamt für Landwirtschaft, Lebensmittelsicherheit und Fischerei Mecklenburg-Vorpommern) according to the recommendation of its Ethics Committee (LALLF, 7221.3-1-039/16) and conducted in compliance with the ARRIVE guidelines, the German animal welfare law, the German guidelines for animal welfare and the EU Directive 2010/63/EU.

#### Middle cerebral artery occlusion (MCAO)

Mice underwent transient MCAO (tMCAO) of the left middle cerebral artery with a filament, which has been described previously^24^. A silicon-coated filament was introduced into the common carotid artery and advanced along the internal carotid artery to the origin of the middle cerebral artery and withdrawn after 20 min.

Exclusion criteria were unsuccessful stroke induction or non-middle cerebral artery territory ischemia based on brain MRI at day one. In addition, well-being of animals was scored and mice were euthanatized before the end of the study in case they reached the humane endpoint score. Only animals that reached the experimental endpoint were included in the study.

#### Orthotopic glioblastoma mouse model

The cell line GL261 was implanted into the right hemisphere of mice brain following a protocol described by Fink et al.^25^. Only animals that showed tumor growth on MRI at the end of the study were included.

### Magnetic resonance imaging

**Figure 2:**
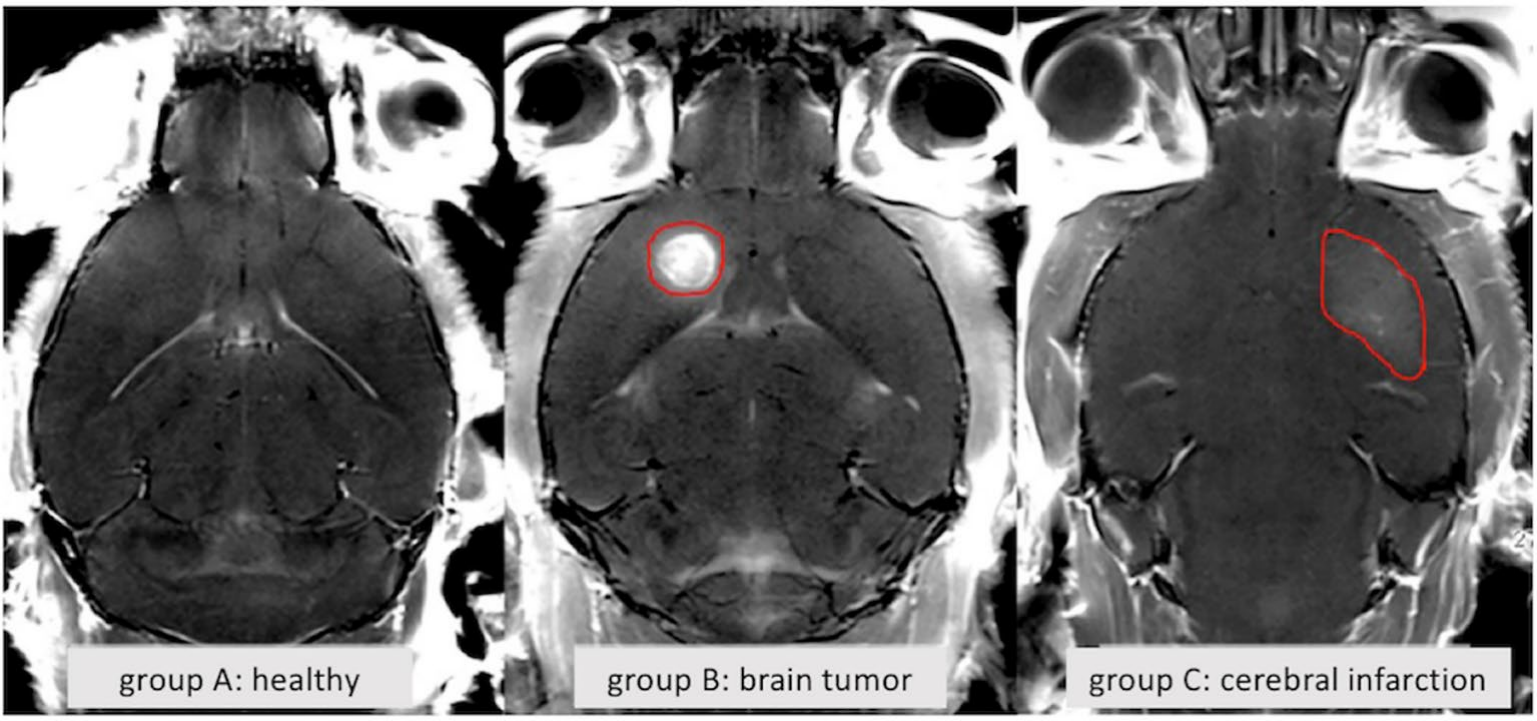
Contrast enhanced T1 weighted MRI of control group and mice after induction of glioblastoma or infarction, respectively. In healthy mice, the contrast agent cannot pass the blood–brain barrier. In case of cerebral tumor or infarction, however, the barrier is disrupted, resulting in enhancement of damaged tissue (red marking).

For intravenous injections and MR acquisition, anesthetized animals were placed inside a 7 Tesla Bruker Clinscan 70/30 system with a maximum gradient of 290 mT/m (Bruker BioSpin GmbH, Germany). Measurements were performed using a mice brain coil (2 × 2) and the following scan parameters: T1-weighted spin echo, repetition time (TR) 500 ms, echo time (TE) 12 ms, flip angle 90°, number of slices 14, slice thickness 0,7 mm, gap 0, field of view (FoV) 25 × 25 mm, resolution 320 × 320 (interpolates to 640 × 640). Additionally, the MCAO group received diffusion weighted imaging at day 2 after experimental stroke induction to assess stroke outcome using the parameters: TR 9000 ms, TE 85 ms, number of slices 10, slice thickness 0,7 mm, gap 0, FoV 35 × 35 mm, resolution 128 × 128 (interpolated 256 × 256). In the glioblastoma group the following additional images were acquired at the end of the study to record enhancement of tumor tissue: T2-weighted turbo spin echo, TR 3080 ms, TE 46 ms, number of slices 18, slice thickness 0.5 mm, gap 0, FoV 25 × 25 mm, resolution 256 × 256. Figure 2 shows MR image examples of control and mice after induction of tumor or infarction, respectively.

### Image analysis

Image analysis was performed using Osirix version 4.6 (Bernex, Switzerland) by 2 independent and blinded observers. Mean signal intensity was quantified by placing a region-of-interest (ROI) on non-contrast MR images at baseline and the last examination in the thalamus, globus pallidus, pons, dentate nucleus, white matter and gray matter. Normalization was undertaken by measuring signal intensity of the intraventricular cerebrospinal fluid (CSF). Globus pallidus-to-thalamus and dentate nucleus-to-pons ratios were determined and compared between baseline and last MR examination as well as among the different groups.

### Laboratory analysis

#### Gadodiamide quantification

Gadodiamide complex was quantified in murine brain tissue after homogenization (1:10 with water) using a liquid chromatography coupled to tandem mass spectrometry (LC–MS/MS)^26^.

MS/MS analysis was done in the positive multiple reaction monitoring mode by monitoring the m/z transitions 573.4/511.5, 573.4/467.4 and 573.4/336.6 for gadodiamide and 366.2/114.1 and 366.2/208.2 for amoxicillin. The method was validated between 5 and 1000 nmol/L and was shown to be of adequate specificity, precision and accuracy (± 15% relative error of the nominal values).

#### Gadolinium quantification

The total gadolinium amount was measured by inductively coupled plasma-mass spectrometry (ICP-MS) from the same homogenized brain samples that were used for gadodiamide quantification. Analytical precision was checked by spiking samples from untreated animals with amounts of gadodiamide that represented the range of the expected gadolinium concentration and parallel processing of these samples. The following isotope abundances were monitored: Li-7; Y-89; Ce-140; Tl-205; Gd-155; Gd-156; Gd-157; Gd-158 and Gd-160.

Signals for gadolinium isotopes were corrected against a linear interpolation of the intensities of the four internal standard isotopes and the obtained values were converted to concentrations by means of the external calibration. The whole workflow was repeated in technical triplicates at differing days and the average concentration is reported together with its standard deviation.

### Statistical analysis

Stratified by group (healthy, glioblastoma, infarction) signal intensities before and after tenfold injection of gadodiamide were reported as means and standard deviations for control and contrast groups. Differences in signal intensities between baseline and follow-up examinations were compared by Wilcoxon-signed rank tests within each of the six groups. Furthermore, differences in signal intensities were compared between contrast and control groups by linear regression models in healthy, glioblastoma, and infarction mice separately.

A value of P < 0.05 was considered statistically significant. Statistical analyses were performed with Stata 16.1 (Stata Corporation, College Station, TX, USA).

## Results

### MR signal enhancement

Mean signal intensity values in the three mice groups at initial and final examination for non-contrast and gadodiamide groups are given in Table 1. There was no significant difference in mean signal intensities before and after tenfold injection of gadodiamide in the animal groups receiving contrast agent within thalamus (p_A_ = 0.820; p_B_ = 0.359; p_C_ = 0.164), pallidum (p_A_ = 0.570; p_B_ = 0.910; p_C_ = 0.203), as well as white matter (p_A_ = 0.734; p_B_ = 0.164; p_C_ = 0.820), whereas measurements in the pons and dentate nucleus showed significantly increased signal intensity for the glioblastoma group (p_pons_ = 0.008, p_dn_ = 0.004) (Table 2). Calculation of the dentate nucleus-to-pons and globus pallidus-to-thalamus SI ratios neither showed a significant increase between baseline and follow-up examination for the healthy mice, glioblastoma or infarction group with and without gadodiamide injection (Table 2).

**Table 1:** Mean signal intensity values in the three mice groups healthy (group A), glioblastoma (group B) and infarction (group C) at initial examination (T_0_) and after tenfold injection of gadodiamide (T_10_).

	Group A control		Group A contrast	
	T_0_	T_10_	T_0_	T_10_
Thalamus	282.7 (± 30.1)	269.1 (± 37.0)	267.5 (± 15.3)	264.7 (± 17.8)
Pallidum	265.1 (± 21.3)	260.0 (± 30.5)	263.9 (± 16.9)	259.1 (± 20.2)
Pons	264.9 (± 34.7)	246.6 (± 30.7)	260.7 (± 23.6)	241.1 (± 21.4)
Dentate nucleus	260.5 (± 37.7)	249.9 (± 33.8)	256.9 (± 26.1)	251.5 (± 23.3)
White matter	325.7 (± 42.2)	318.3 (± 38.7)	324.0 (± 33.2)	317.4 (± 33.4)
DN/pons	0.98 (± 0.07)	1.01 (± 0.04)	0.99 (± 0.06)	1.04 (± 0.07)
GP/thalamus	0.94 (± 0.05)	0.97 (± 0.08)	0.99 (± 0.06)	0.98 (± 0.06)

	Group B control		Group B contrast	
	T_0_	T_10_	T_0_	T_10_
Thalamus	269.8 (± 39.1)	259.0 (± 37.6)	271.6 (± 13.2)	250.8 (± 38.1)
Pallidum	269.9 (± 40.5)	263.8 (± 35.4)	274.9 (± 16.5)	258.8 (± 30.7)
Pons	258.0 (± 44.3)	236.4 (± 38.2)	256.8 (± 14.2)	223.3 (± 39.7)
Dentate nucleus	267.8 (± 53.3)	233.5 (± 43.0)	264.7 (± 18.0)	224.9 (± 47.4)
White matter	310.5 (± 48.2)	314.0 (± 49.3)	329.3 (± 20.7)	291.8 (± 52.7)
DN/pons	1.03 (± 0.04)	0.99 (± 0.08)	1.03 (± 0.04)	1.00 (± 0.06)
GP/thalamus	1.00 (± 0.03)	1.02 (± 0.04)	1.01 (± 0.03)	1.04 (± 0.05)

	Group C control		Group C contrast	
	T_0_	T_10_	T_0_	T_10_
Thalamus	259.3 ± 17.1	279.2 (± 12.3)	237.1 ± 30.6	261.1 (± 40.2)
Pallidum	262.0 ± 25.8	278.3 (± 12.8)	237.1 ± 30.6	265.7 (± 41.4)
Pons	258.5 ± 12.0	258.8 (± 13.1)	234.6 ± 31.4	240.0 (± 33.9)
Dentate nucleus	269.7 ± 20.9	267.7 (± 36.7)	241.4 ± 39.4	243.7 (± 39.9)
White matter	315.3 ± 19.3	323.7 (± 24.0)	299.7 ± 40.4	308.4 (± 49.8)
DN/pons	1.04 ± 0.04	1.03 (± 0.10)	1.02 ± 0.05	1.01 (± 0.05)
GP/thalamus	1.01 ± 0.04	1.00 (± 0.05)	1.02 ± 0.04	1.02 (± 0.05)

**Table 2:** Comparison of signal intensities (p-values) in the three mice groups (healthy A, glioblastoma B, infarction C) between initial examination and after tenfold injection of gadodiamide.

	Group A	Group B	Group C
Thalamus	0.820	0.359	0.164
Pallidum	0.570	0.910	0.203
Pons	**0.039**	**0.008**	0.496
Dentate nucleus	0.734	**0.004**	0.734
White matter	0.734	0.164	0.820
DN/pons	0.250	0.250	0.570
GP/thalamus	0.734	0.129	0.426

p-values derived from signed rank tests.

Significant values are in bold.

When comparing control and contrast groups within each category (healthy, glioblastoma and infarction) at last MR examination (that is after 10 time gadolinium injection for the contrast group), no significant increase in signal intensity could be seen for any neuronal structure (Table 3). Glioblastoma mice receiving tenfold gadolinium injection showed only a significantly higher SI for the globus pallidus-to-thalamus ratio compared with healthy contrast mice (p = 0.033). For the infarction group no significant SI increase could be detected compared to healthy mice after contrast application (Table 3).

**Table 3:** Signal intensities (p-values) at last MR examination in control groups (without Gadolinium injection) compared to mice receiving tenfold Gadolinium administration (healthy A, glioblastoma B, infarction C).

	A_control_ vs A_Gd_	B_control_ vs B_Gd_	C_control_ vs C_Gd_	A_Gd_ vs B_Gd_	A_Gd_ vs C_Gd_
Thalamus	0.775	0.629	0.843	0.367	0.814
Pallidum	0.950	0.634	0.793	0.983	0.667
Pons	0.711	0.553	0.801	0.229	0.937
Dentate nucleus	0.930	0.817	0.858	0.145	0.665
White matter	0.965	0.119	0.990	0.208	0.658
DN/pons	0.330	0.678	0.957	0.187	0.318
GP/thalamus	0.771	0.879	0.997	**0.033**	0.164

Significant values are in bold.

**Figure 3:**
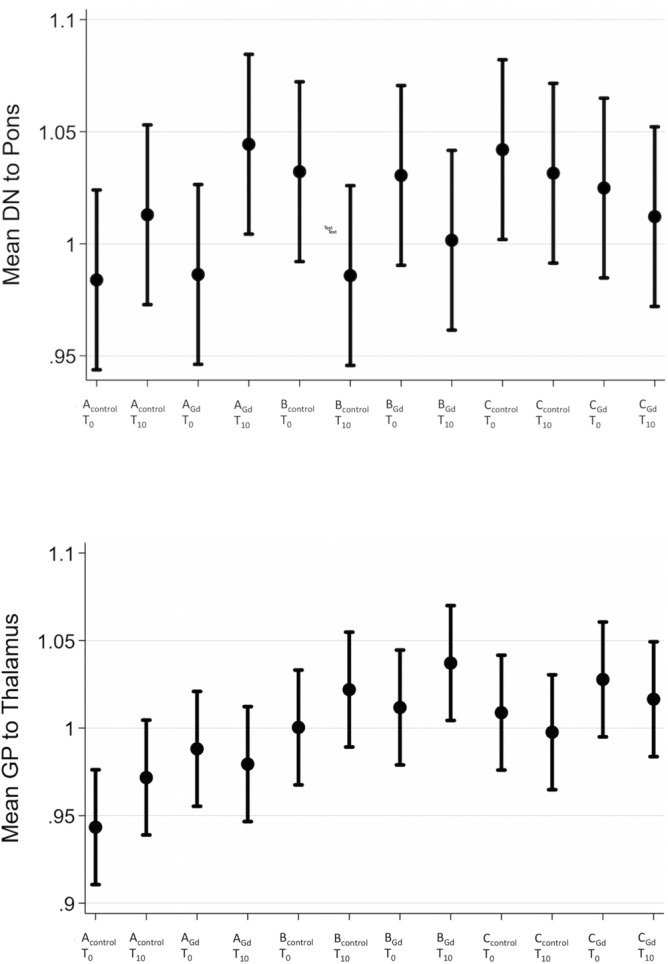
Mean globus pallidus-to-thalamus and dentate nucleus-to-pons ratios and 95%-interval at baseline (T_0_) and last MR examination (T_10_) among the different groups A healthy mice, B glioblastoma mice and C cerebral infarction mice.

Percentage changes in relative signal intensities for dentate nucleus-to-pons and globus pallidus-to-thalamus ratios in the contrast and control groups at initial and last MR examinations are depicted in Fig. 3.

Comparing contrast group and control group within each category (healthy, glioblastoma, infarction), there were no statistically significant differences in changes of the signal intensities between the first and last examination in thalamus, p = 0.576; pallidum, p = 0.984; pons, p = 0.944; dentate nucleus, p = 0.748; white matter, p = 0.967 for healthy mice (group A). Same results were seen in glioblastoma mice (group B): thalamus, p = 0.692; pallidum, p = 0.694; pons, p = 0.633; dentate nucleus, p = 0.850; white matter, p = 0.231, as well as mice with cerebral infarction (group C): thalamus, p = 0.827; pallidum, p = 0.797; pons, p = 0.774; dentate nucleus, p = 0.864; white matter, p = 0.989. Likewise, no significant change could be detected comparing contrast and control groups for both ratios over time (p_A dn-to-pons_ = 0.519, p_A gp-to-thal_ = 0.161; p_B dn-to-pons_ = 0.657, p_B gp-to-thal_ = 0.873; p_C dn-to-pons_ = 0.959; p_C gp-to-thal_ = 0.997).

### Inter- and intraobserver reliability

To evaluate data quality, quality assurance of all records was performed using interobserver reliability with Bland–Altman analysis, including the calculation of the interclass correlation coefficient, showing acceptable outcomes with thalamus 0.62, globus pallidus 0.74, pons 0.63 and dentate nucleus 0.73.

### Gadodiamide/gadolinium quantification

Quantitative analysis showed a significantly higher cerebral concentration of gadodiamide after 10 times contrast agent injection in the glioblastoma group (0.527 nmol/g) compared to the control group (0.232 nmol/g) without blood–brain barrier disturbance (p = 0.001). Although the infarction group also showed a higher amount of gadodiamide (0.356 nmol/g), this was not significant (p = 0.106).

The total amount of gadolinium in mouse brains treated with gadodiamide was also highest in group B with 5.03 nmol/g (group A: 4.18 nmol/g; group C: 4.81 nmol/g), however, without being statistically significant (p_B_ = 0.066; p_C_ = 0.165).

Calculation of the chelate-to-total gadolinium ratio revealed a significantly higher value in glioblastoma mice (p = 0.013), but not for mice with infarction (p = 0.918) compared to healthy controls.

## Discussion

Alterations of the blood–brain barrier function, as in the course of vascular or parenchymal injuries, could facilitate the accumulation of gadolinium in the cerebrum. This study investigates the impact of blood–brain barrier disturbance—caused by cerebral infarction or malignant brain tumor—on intracerebral residual gadolinium in a mouse model. We found that multiple application of the linear contrast agent gadodiamide (about fourfold the human standard dose) leads to cerebral gadolinium deposition without correlate in MR imaging.

Gadolinium presence has been proposed in clinical studies on the basis of T1 hyperintensities observed in the globus pallidus and dentate nucleus^16,17,27^. However, previously reported T1 abnormalities after repeated GBCA injection have to be evaluated with caution, since many patients undergoing multiple contrast-enhanced MR examinations have a history of neoplastic disease, multiple sclerosis, brain radiation etc., which frequently show high signal intensities on unenhanced T1-weighted images^28–36^. Such signaling may, therefore, potentially be associated with the underlying illnesses rather than contrast agent application. In our study, we could not detect a convincing cerebral increase in MR signal intensity after tenfold administration of the linear chelate gadodiamide in mice with blood–brain barrier disturbance. Nonetheless, we could chemically detect the presence of both, gadodiamide and gadolinium in the brain samples at the experimental endpoint regardless of the blood–brain barrier status.

Preclinically, several animal studies have already given insides into GBCA kinetics, toxicity and chemical form of neuronal residual gadolinium^37–40^. In two different publications the presence of a soluble macromolecular fraction was identified in different regions of the rat brain in addition to intact soluble gadodiamide^38,40^. Gianlio et al.^38^, reported that the relaxivity of this macromolecular species was around 100 mM/s, which might be high enough to produce the T1 hyperintensities observed in human studies of residual gadolinium.

However, studies investigating animals with neuronal pathologies remain scarce. One study by Arena et al.^41^ evaluated the effect of repeated administrations of gadodiamide in rats with cerebral chronic hypoperfusion as a model for blood–brain barrier alteration seen in neurodegenerative diseases and the aging brain in general. Here, ex vivo tissue analysis performed by ICP-MS showed greater gadolinium presence in subcortical regions. This is confirmed by our findings where the concentration of cerebral gadodiamide as well as total amount of gadolinium was higher in the mouse group with induced infarction compared to the control group. In addition, we detected higher gadodiamide and total gadolinium concentration in glioblastoma mice, which was significant for gadodiamide. However, Arena et al. reported on T1 hyperintensities in the dentate nucleus and hippocampus, which we could not replicate in our study. The observed discrepancies may be due to a divergent injection regimen—in our study gadodiamide was injected 10 times over a time span of 20 days, whereas Arena et al. performed 22 administrations over 7 weeks. Another explanation for the missing SI increase in our study after gadolinium administration could be the presence of gadolinium in an MRI-silent form (e.g. in the form of insoluble precipitates in non-enhancing regions but as chelated or bound to macromolecules in enhancing regions^42^.

Previous studies proposed an involvement of the so called glymphatic system for the uptake, distribution and elimination of gadodiamide and other GBCAs to the brain^43,44^. Taoka et al.^45^ examined the influence of the glymphatic system on kinetic and distribution of intravenously injected gadodiamide in the rat brain and found that the cerebrospinal fluid is one potential pathway of GBCAs entry into the brain. The disruption of the blood–brain barrier, in our opinion, constitutes a different route for the uptake and perhaps also the clearance of GBACs. Orthotopic glioblastoma and middle cerebral artery occlusion are animal disease models for imaging blood–brain barrier disturbance in mice^21^, with the difference that glioblastoma cause an increasing blood–brain barrier permeability, whereas MCAO leads only to a transient BBB disturbance^22^—longitudinal studies after a transient 20 min. MCAO suggested a BBB disturbance peaking at day 7 and resolving at day 14 after ischemia^46^.

Recently, Strzeminska et al. reported that only as little as 12–13% of the total gadolinium in rat brain samples was recovered in the originally administered gadodiamide form while the vast majority of the element underwent ligand exchange or chemical transformation^47^. In our work, 10.5% of the gadolinium in glioblastoma mice and 5.6% or 7.4% of the gadolinium in healthy and MCAO mice, respectively, was present as gadodiamide. The increase in stable gadodiamide and total gadolinium amount with increasing blood–brain border disturbance may indicate accelerated uptake of the complex into brain structures at a limited transformation rate. However, it was suggested that gadodiamide is less stable in the brain than in blood plasma and thus repeated uptake and readsorption of gadodiamide over longer periods would lead to accumulation in the brain under impaired blood–brain border conditions^48^. In fact, a larger fraction of the injected gadolinium accumulated in the brain in mice with malignant brain tumor.

The increased gadolinium accumulation seen in conditions of blood–brain barrier disturbance without imaging correlate in MRI, are supposedly caused by gadodiamide complex/total gadolinium concentrations, which although detectable via a sensitive method like mass spectrometry are too low to cause visible signal change in MRI.

In conclusion, this study (chemically) detected neuronal gadolinium accumulation after repeated administration of gadodiamide due to blood–brain barrier dysfunction in infarction and glioblastoma mouse models, without measurable imaging correlate in MR. This supports recent suggestions of a restricted usage of linear contrast agents in favor of macrocyclic ones.

## Data Availability

The datasets used and/or analysed during the current study are available from the corresponding author on reasonable request.

## Abbreviations

BBB: Blood–brain barrier
CSF: Cerebrospinal fluid
Dn-to-pons: Dentate nucleus-to-pons
FoV: Field of view
GBCA: Gadolinium-based contrast agent
gp-to-thal: Globus pallidus-to-thalamus
ICP-MS: Inductively coupled plasma-mass spectrometry
LALLF: Landesamt für Landwirtschaft, Lebensmittelsicherheit und Fischerei Mecklenburg-Vorpommern
LC–MS/MS: Liquid chromatography coupled to tandem mass spectrometry
MCAO: Middle cerebral arterial occlusion
MRI: Magnetic resonance imaging
NSF: Nephrogenic systemic fibrosis
PES: Polyethersulfone
PFA: Perfluoralkoxylalkan
ROI: Region-of-interest
SI: Signal intensity
TE: Echo time
tMCAO: Transient MCAO
TR: Repetition time
